# Supplementary figures and images for: Limited sensitivity of somatosensory evoked potentials as disease monitoring biomarkers in hereditary spastic paraplegias
Source: PLoS One. 2025 Nov 11;20(11):e0335187. doi: 10.1371/journal.pone.0335187 (PMC12604765; doi:10.1371/journal.pone.0335187)

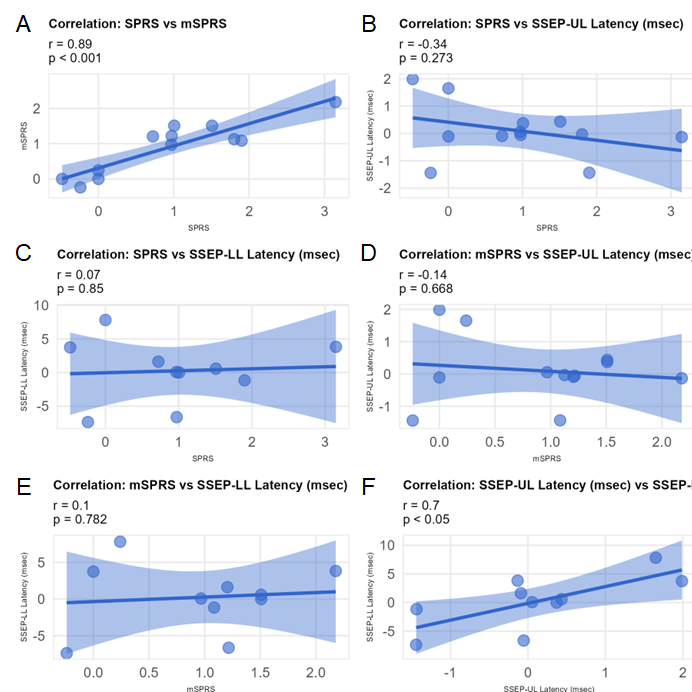

Supplement: S1 Fig — SPRS: Spastic Paraplegia Rating Scale; mSPRS: motor Spastic Paraplegia Rating Scale. SSEP-UL upper limbs somatosensory evoked potential. SSEP-LL lower limbs somatosensory evoked potential. (TIF) [file pone.0335187.s001.tif]

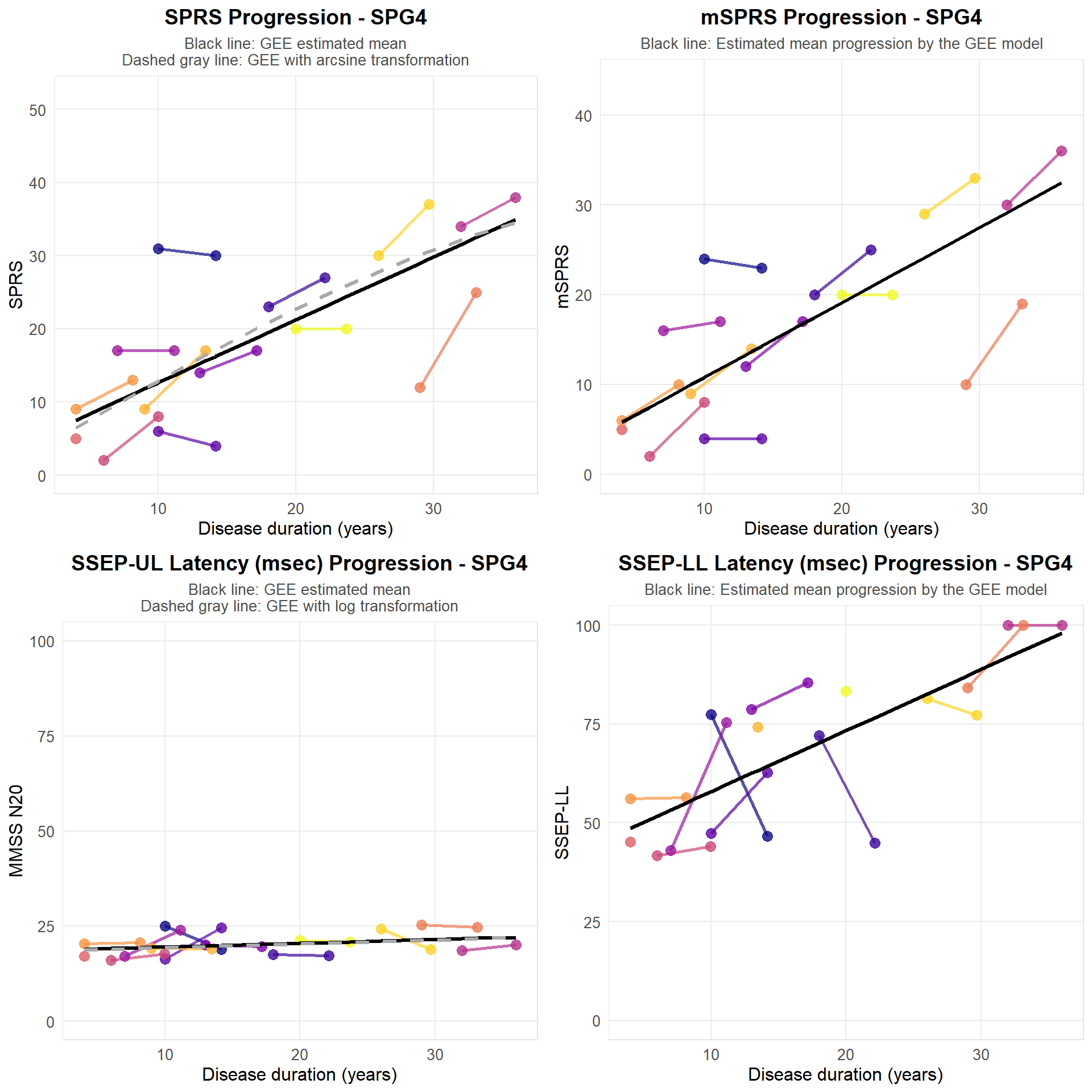

Supplement: S2 Fig — Progression of the A) Spastic Paraplegia Rating Scale (SPRS), B) motor-SPRS (mSPRS), C) latency of the SSEP-Upper Limb (SSEP-UL) and D) latency of the SSEP-Lower Limb (SSEP-LL) over time. Dashed gray lines represent the results after applying arcsine or logarithmic transformations, as shown in the S2 Table. GEE Generalized Estimating Equations. (TIF) [file pone.0335187.s002.tif]
