# Supplementary material for: Limited sensitivity of somatosensory evoked potentials as disease monitoring biomarkers in hereditary spastic paraplegias
Source: PLoS One. 2025 Nov 11;20(11):e0335187. doi: 10.1371/journal.pone.0335187 (PMC12604765; doi:10.1371/journal.pone.0335187)
Supplement: S2 Table — (DOCX) [file pone.0335187.s004.docx]

**Supplementary Table 2 - Progression by Follow-up Time in the SPG4 Subgroup**

| **Variable** | **Mean (CI 95%)** | **SRM (CI 95%)** | **p-value** |
| --- | --- | --- | --- |
| SPRS | 0.94 [0.28 to 1.61] | 0.90 [0.47 to 1.71] | 0.010 |
| mSPRS | 0.90 [0.43 to 0.74] | 1.22 [0.73 to 2.68] | < 0.001 |
| SSEP-UL Latency (msec) | 0.10 [-0.54 to 0.74] | 0.10 [-0.55 to 0.68] | 0.737 |
| SSEP-LL Latency (msec) | 0.25 [-3.05 to 3.55] | 0.05 [-0.56 to 0.92] | 0.869 |

SPRS: Spastic Paraplegia Rating Scale; mSPRS: motor Spastic Paraplegia Rating Scale; SRM, standardized response mean; SSEP-UL upper limbs somatosensory evoked potential. SSEP-LL lower limbs somatosensory evoked potential
