# Supplementary material for: Limited sensitivity of somatosensory evoked potentials as disease monitoring biomarkers in hereditary spastic paraplegias
Source: PLoS One. 2025 Nov 11;20(11):e0335187. doi: 10.1371/journal.pone.0335187 (PMC12604765; doi:10.1371/journal.pone.0335187)
Supplement: S3 Table — (DOCX) [file pone.0335187.s005.docx]

**Supplementary Table 3 – Disease progression modeled according to the disease duration with data transformation**

| **Variable** | **Estimate** | **Standard Error** | **Statistic** | **Mean (CI 95%)** | **p-value** |
| --- | --- | --- | --- | --- | --- |
| SPRS arcsine | 0.0264 | 0.0039 | 45.2845 | 0.0187 | 0.0340 |
| SSEP-UL log Latency (msec) | 0.0076 | 0.0029 | 6.4453 | 0.0017 | 0.0135 |

SPRS: Spastic Paraplegia Rating Scale; SSEP-UL upper limbs somatosensory evoked potential.
