# Supplementary material for: Limited sensitivity of somatosensory evoked potentials as disease monitoring biomarkers in hereditary spastic paraplegias
Source: PLoS One. 2025 Nov 11;20(11):e0335187. doi: 10.1371/journal.pone.0335187 (PMC12604765; doi:10.1371/journal.pone.0335187)
Supplement: S4 Table — (DOCX) [file pone.0335187.s006.docx]

**Supplementary Table 4** - **Disease progression modeled according to the disease duration in the SPG4 subgroup**

| **Variable** | **Estimate** | **Standard Error** | **Statistic** | **Mean (CI 95%)** | **p-value** |
| --- | --- | --- | --- | --- | --- |
| SPRS | 0.858 | 0.1376 | 38.8495 | 0.59 to 1.23 | < 0.001 |
| mSPRS | 0.834 | 0.1254 | 44.2148 | 0.59 to 1.08 | < 0.001 |
| SSEP-UL Latency (msec) | 0.164 | 0.0694 | 5.602124 | 0.03 to 0.3 | 0.111 |
| SSEP-LL Latency (msec) | 1.546 | 0.1828 | 71.5180 | 1.19 to 1.90 | < 0.001 |

SPRS: Spastic Paraplegia Rating Scale; mSPRS: motor Spastic Paraplegia Rating Scale. SSEP-UL upper limbs somatosensory evoked potential. SSEP-LL lower limbs somatosensory evoked potential
